# Association between triglyceride-glucose index and risk of cardiovascular disease among postmenopausal women

**DOI:** 10.1186/s12933-023-01753-3

**Published:** 2023-01-30

**Authors:** Qian Liu, Fei Si, Zhou Liu, Yuntao Wu, Jing Yu

**Affiliations:** 1 Lanzhou University Second Hospital, Lanzhou University, Lanzhou, China; 2 Department of Cardiology, Kailuan General Hospital, 57 Xinhua East Rd., Tangshan, 063000 Hebei China; 3 Department of Cardiology, Lanzhou University Second Hospital, No. 82 Cuiyingmen, Lanzhou, 730000 Gansu China

**Keywords:** Triglyceride-glucose index, Cardiovascular disease, Postmenopausal, Prospective cohort study

## Abstract

**Objective:**

We aimed to examine the association of triglyceride-glucose index (TyG) with risk for cardiovascular disease (CVD) among postmenopausal women.

**Methods:**

A total of 7741 participants met the inclusion criteria, and were included in the analysis. The TyG index was calculated as ln (triglyceride [mg/dL] × fasting blood glucose [mg/dL]/2). The participants were classified into four groups by the quartiles of TyG index, and the Q1 group was used as the reference group. The cumulative incidence of CVD for the groups were compared using the Kaplan–Meier curves. The association between the TyG index and risk of CVD among postmenopausal women was assessed by the Cox proportional hazards models (hazard ratio [HR], 95% confidence intervals [CI]).

**Results:**

During a median follow-up of 12 years, a total of 383 (4.95%) participants developed incident CVD. After adjusting for potential confounding factors, a high baseline TyG index (Q4 group) was associated with higher future risk of CVD, the HR (95% CI) of CVD risk was 1.70 (1.21–2.38) in Q4 group compared with the Q1 group. Subgroup analyses showed the Q4 group was significantly associated with the risk of CVD, regardless of age at menopause (younger than 50 years; 50 years and older) and obesity status.

**Conclusions:**

Higher TyG index at baseline as a marker of insulin resistance (IR), is associated with higher risk of future CVD among postmenopausal women. The TyG index may serve as a simple and easy marker for early identification of high-risk individuals in the postmenopausal women.

**Supplementary Information:**

The online version contains supplementary material available at 10.1186/s12933-023-01753-3.

## Introduction

The *Lancet* women and cardiovascular disease Commission reported that the prevalence of cardiovascular disease (CVD) increased by 7.5% in Chinese women during the past the 30 years. CVD is the leading cause of death in women worldwide, and accounts for nearly 35% of all global deaths in women [1]. It is critically significant for a focus on women’s health, exploring influencing factors and preventive strategies of CVD to delay and lower risk for CVD, thus alleviating the global burden of CVD. Several studies have showed that insulin resistance (IR), which is defined as a reduced efficiency of promoting glucose uptake to tissue and inhibiting hepatic glucose production, is a significant risk factors for CVD [2–4]. The menopausal transition is associated with an increase in IR and contributes to deteriorate cardiometabolic transition. Therefore, menopausal transition is associated with increased risk for cardiometabolic disease such as CVD [5, 6]. Focusing on IR in postmenopausal women and implementing early intervention strategies may be key for postmenopausal women to lower the risk of future CVD. A prospective cohort studies including 15,288 postmenopausal women indicates that IR, which is assessed by homeostasis model assessment of IR (HOMA-IR) based on fasting glucose and insulin levels, is associated with the risk for the development of CVD [7]. However, IR measured by insulin concentrations is not measured routinely in clinical practice, which is not suitable for daily large-scale clinical studies. Previous studies had found triglyceride–glucose (TyG) index was suggested as a non-invasive, simple, reliable and effective surrogate marker of IR [8, 9]. And TyG index have been shown to be associated with higher risk of CVD in the general population [10]. However, to the best of our knowledge, no relevant studies have investigated the association between the TyG index and CVD in postmenopausal women. Thus, we aimed to investigated the association between the TyG index and future CVD among postmenopausal women based on Kailuan Study.

## Methods

### Study population and design

The Kailuan study, an ongoing prospective cohort study, was initiated in 2006 years and conducted follow-up every 2 years and biannually thereafter [11, 12]. A total of 101,510 participants (81,110 men and 20,400 women) aged 18–98 years old completed the first health examination and collected related information in the Kailuan general hospital and its 10 affiliated hospitals.

A total of 9407 participants who participated in the health examinations from 2006 to 2015 and with complete menopausal information were included. We excluded 1666 participants who could meet the following criteria: (1) 27 participants with history of cardiovascular disease; and (2) 1639 participants with missing data of triglyceride (TG) and/or fasting blood glucose (FBG) at baseline. Therefore, a total of 7741 participants were finally included in the analysis (Additional file 1: Figure S1).

This study was approved by the ethics committees of Kailuan General Hospital (Approval Number: 2006-05) and informed consent had been obtained from all the participants. The study was performed according to the guidelines of the Helsinki Declaration.

### Data collection and definitions

Information on participants’ sociodemographic characteristics, history of CVD, medication use, and lifestyle, including age, gender, smoking status, drinking status, physical activity, educational status, menopausal information, and so on, were collected using questionnaires by professionally trained employees, details are provided in reference [13]. Smoking and drinking status were classified into current, former or never smokers. Physical activity was defined as exercising at least 4 times a week, with at least 20 min each time. Salt intake was classified as < 6 g/day, 6–12 g/day, and > 12 g/day according to questions related to salt intake. Education status was categorized as illiterate, primary school, junior high school, high school, and college degree or above. Body mass index (BMI, kg/m^2^) was calculated as weight (kg) divided by height^2^ (m^2^). Systolic blood pressure and diastolic blood pressure were measured 3 times repeatedly by qualified medical personnel using standard methods, and the results were averaged [14]. Menopausal information included menopausal or not, age at menarche, age at menopause, and menstrual cycle. Self-reported menopausal status was categorized as postmenopausal and premenopausal, whether it was natural or surgical induced.

Blood samples were drawn on the day of physical examination after at least 8 h of fasting. The biochemical index, including total cholesterol (TC), FBG, TG, low density lipoprotein cholesterol (LDL-C), high density lipoprotein cholesterol (HDL-C), high sensitive C-reactive protein (Hs-CRP), and so on, were measured on the Hitachi automatic biochemical analyzer (Hitachi 747, Tokyo, Japan) [15].

We defined hypertension as self-reported use of antihypertensive medication or a BP measurement ≥ 140/90 mm Hg (1 mm Hg = 0.133 kPa) or a self-reported history [16].

Diabetes was defined as FBG ≥ 7 mmol/L or a self-reported use of anti-diabetic drugs or medical history of diabetes [17]. Dyslipidemia was defined as non-HDL cholesterol level ≥ 4.9 mmol/L or self-reported use of lipid-lowering drugs.

### Assessment of TyG index

The TyG index was calculated as ln (TG [mg/dL] × FBG [mg/dL]/2) [9]. The TyG index at baseline was calculated from the date on TG and FBG first measured from 2006 to 2015 years. The participants were classified into four groups (Q1, Q2, Q3, Q4) by the quartiles of TyG index, and the Q1 group was used as the reference group.

### Outcomes and follow-up

This study took the date of first physical examination in postmenopausal women as baseline, and the date of follow-up was 31 December 2020. The study endpoint was the first incident CVD, death or 31 December 2020, whichever came first. The CVD included myocardial infarction (MI) and stroke according to the *World Health Organization* criteria [18, 19]. The CVD events were diagnosed by trained medical staff through the hospital’s electronic medical records review and medical insurance system. Information on death was collected from death certificates from state vital statistics offices and discharge electronic medical records.

### Statistical analysis

Continuous variables of normal distribution were described using mean and standard deviation (SD). Skewed variables were presented as median and interquartile range (M [P25, P75]). Categorical variables were expressed by the number of case and percentage. The cumulative incidence of CVD for the groups were compared using the Kaplan–Meier curves. The Cox proportional hazards models were applied to assess the association between the TyG index and risk of CVD among postmenopausal women. Model 1 was adjusted for age at baseline; model 2 included the covariates in model 1, BMI, education status, current drinker, current smoker, and physical activity; model 3 included the covariates in model 2, salt intake, LDL-C, HDL-C, hypertension, and diabetes; model 4 further adjusted for age at menopause.

We conducted several stratified and sensitivity analyses to test the stability of our results. First, we conducted analyses stratified by hypertension status (yes or no), age at menopause (< 50 years or ≥ 50 years), diabetes status (yes or no), BMI level (< 28 kg/m^2^ or ≥ 28 kg/m^2^), and evaluated the interaction via a likelihood ratio test. Second, we carried out sensitivity analysis after excluding those who used anti-diabetic drugs, antihypertensive drugs, or lipid-lowering drugs. Another sensitivity analyses were conducted after excluding participants with diabetes or severe dyslipidemia. Sensitivity analyses were also conducted by subtypes of CVD (MI and stroke).

All statistical analyses were conducted using SAS 9.4 (SAS Institute, Inc., Cary, NC, USA), and a two-tailed P < 0.05 was considered statistically significant.

## Results

### Baseline characteristics

The baseline characteristics of the participants by the quartiles of the TyG index are shown in Table 1. A total of 7741 participants were included in the study, with a mean age of 55.49 (SD = 6.24) years at baseline and 50.19 (SD = 3.41) years at menopause.

**Table 1:** Baseline characteristics of participants according to quartiles of TyG index among postmenopausal women

Characteristic	Quartiles of TyG index			
	Q1(< 8.26)	Q2(8.26–8.62)	Q3(8.62–9.07)	Q4(9.07–12.78)
Number of participants	1995	1914	1911	1921
Age, years	54.48 ± 6.01	55.24 ± 6.36	55.89 ± 6.29	56.40 ± 6.15
Age at menopause, years	49.93 ± 3.43	50.17 ± 3.35	50.23 ± 3.49	50.44 ± 3.36
BMI, kg/m^2^	23.84 ± 3.40	24.87 ± 3.50	25.83 ± 3.44	26.36 ± 3.53
SBP, mmHg	123.40 ± 18.34	128.50 ± 19.58	132.84 ± 20.43	135.63 ± 20.28
DBP, mmHg	78.76 ± 10.12	81.03 ± 10.44	83.25 ± 10.76	84.15 ± 10.80
FBG mmol/L	4.80 ± 0.72	5.10 ± 0.82	5.38 ± 1.05	6.61 ± 2.53
HDL-C, mmol/L	1.74 ± 0.86	1.62 ± 0.99	1.56 ± 0.41	1.46 ± 0.46
TC, mmol/L	4.92 ± 0.94	5.19 ± 0.95	5.48 ± 1.75	5.45 ± 1.39
LDL, mmol/L	2.24 ± 1.15	2.49 ± 0.99	2.59 ± 1.07	2.53 ± 1.11
TG, mmol/L	0.77(0.63, 0.90)	1.16(1.03, 1.29)	1.63(1.43, 1.85)	2.65(2.16, 3.51)
Hs-CRP, mg/L	0.93(0.38, 2.90)	1.10(0.42, 3.00)	1.50(0.60, 3.50)	1.90(0.80, 4.62)
Tyg index	7.94 ± 0.27	8.44 ± 0.10	8.83 ± 0.13	9.55 ± 0.45
Current drinker, n(%)	97(4.86)	79(4.13)	80(4.19)	74(3.85)
Current smoker, n(%)	26(1.30)	37(1.93)	31(1.62)	47(2.45)
Physical activity, n(%)	372(18.65)	351(18.34)	352(18.42)	379(19.73)
Education status, n(%)				
Illiterate	8(0.40)	9(0.47)	7(0.37)	8(0.42)
Primary school	120(6.02)	116(6.06)	133(6.96)	169(8.80)
Junior high school	1346(67.47)	1328(69.38)	1284(67.19)	1289(67.10)
High school	375(18.80)	349(18.23)	381(19.94)	359(18.69)
College degree or above	146(7.32)	112(5.85)	106(5.55)	96(5.00)
Salt intake, n(%)				
< 6 g/day	279(13.98)	262(13.69)	269(14.08)	247(12.86)
6–12 g/day	1607(80.55)	1538(80.36)	1488(77.86)	1545(80.43)
> 12 g/day	109(5.46)	114(5.96)	154(8.06)	129(6.72)
Anti-diabetic drugs, n(%)	14(0.70)	32(1.67)	64(3.35)	185(9.63)
Antihypertensive drugs, n(%)	166(8.32)	239(12.49)	354(18.52)	391(20.35)
Lipid-lowering drugs, n(%)	18(0.90)	22(1.15)	38(1.99)	54(2.81)

BMI, body mass index; SBP, systolic blood pressure; DBP, diastolic blood pressure; TC, total cholesterol; LDL-C, low density lipoprotein cholesterol; HDL-C, high density lipoprotein cholesterol; TG, triglyceride; Hs-CRP, high sensitive C-reactive protein; FBG, fasting blood glucose

### Association between TyG index and risk of cardiovascular disease among postmenopausal women

**Fig. 1:**
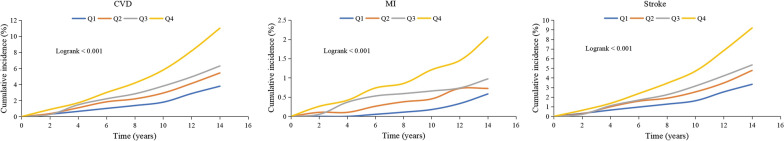
Kaplan–Meier curves of incidence of outcomes according to quartiles of baseline TyG index

During a median follow-up of 12 years, a total of 383 (4.95%) participants developed incident CVD. The Kaplan–Meier survival curves showed the participants in Q4 group had a higher cumulative incidence for CVD compared with those of other groups over follow-up time (log-rank test, P < 0.01; Fig. 1).

Table 2 showed the association between a high baseline TyG index (Q4 group) and future risk of CVD after adjusting for potential confounding factors. In model 1, the HR (95%CI) of CVD risk was 2.76 (2.02–3.75) in Q4 group compared with the Q1 group. After adjusting for variables in model 1 plus BMI, education status, current drinker, current smoker, physical activity at baseline, the Q4 group showed an increased risk of developing CVD (HR [95% CI], 2.40 [1.75–3.29]) compared to the Q1 group. In model 3, the Q4 group had a 70% increased risk of CVD compared with Q1 group. Further adjustment for age at menopause yielded similar results (Q4 HR: 1.70, 95% CI 1.21–2.38).

**Table 2:** Association between TyG index and risk of cardiovascular disease among postmenopausal women

Outcomes	Quartiles of TyG index				P for trend	Per 1 unit increase
	Q1	Q2	Q3	Q4		
Case, n(%)	54(2.71)	77(4.02)	94(4.92)	158(8.22)		
Incidence, per 1000 person-y	2.44	3.65	4.38	7.39		
Model 1	Reference	1.44(1.02–2.04)	1.66(1.19–2.32)	2.76(2.02–3.75)	< 0.01	1.73(1.50–1.98)
Model 2	Reference	1.33(0.94–1.89)	1.50(1.07–2.10)	2.40(1.75–3.29)	< 0.01	1.63(1.41–1.88)
Model 3	Reference	1.25(0.88–1.78)	1.26(0.89–1.78)	1.70(1.21–2.38)	< 0.01	1.31(1.11–1.54)
Model 4	Reference	1.25(0.88–1.78)	1.26(0.89–1.78)	1.70(1.21–2.38)	< 0.01	1.31(1.11–1.54)

Model 1 was adjusted for age at baseline

Model 2 was adjusted for variables in model 1 plus BMI, education status, current drinker, current smoker, physical activity at baseline

Model 3 was adjusted for variables in model 2 plus salt intake, LDL-C, HDL-C, hypertension, diabetes

Model 4 was adjusted for variables in model 3 plus age at menopause

### Subgroup analyses

Table 3 showed the results of the subgroup analyses. Among postmenopausal women, the Q4 group was significantly associated with the risk of CVD, regardless of age at menopause (younger than 50 years; 50 years and older) and BMI level (< 28 and ≥ 28 kg/m^2^). The highest quartile of baseline TyG index was associated with an increased risk of CVD in participants without a history of diabetes mellitus or hypertension. The highest quartile of baseline TyG index was not associated with CVD in participants with a history of diabetes mellitus or hypertension. There was no interaction in the all subgroups.

**Table 3:** Association between TyG index and risk of cardiovascular disease among postmenopausal women in stratified analysis

Variables	Group	Model 2		Model 3		Model 4	
		HR(95% CI)	P _interaction_	HR(95% CI)	P _interaction_	HR(95% CI)	P _interaction_
Age at menopause			0.64		0.65		0.62
< 50 years(n = 2133)	Q4	2.95(1.65–5.28)		1.96(1.05–3.67)		1.97(1.05–3.68)	
≥ 50 years(n = 5608)	Q4	2.24(1.53–3.26)		1.62(1.08–2.42)		1.62(1.08–2.43)	
Hypertension			0.43		0.27		0.27
No(n = 4471)	Q4	2.41(1.45–3.98)		2.02(1.17–3.46)		2.02(1.18–3.46)	
Yes(n = 3270)	Q4	1.93(1.27–2.92)		1.47(0.95–2.28)		1.47(0.95–2.28)	
Diabetes			0.32		0.30		0.30
No(n = 6913)	Q4	1.83(1.29–2.61)		1.61(1.12–2.32)		1.61(1.12–2.32)	
Yes(n = 828)	Q4	2.52(0.58–10.96)		2.41(0.51–11.28)		2.40(0.51–11.29)	
BMI			0.27		0.36		0.36
< 28 kg/m^2^(n = 6210)	Q4	1.96(1.38–2.79)		1.34(0.91–1.96)		1.34(0.91–1.96)	
≥ 28 kg/m^2^(n = 1531)	Q4	4.75(1.90–11.84)		3.66(1.43–9.34)		3.65(1.43–9.33)	

Model 2 was adjusted for age, BMI, education status, current drinker, current smoker, physical activity at baseline

Model 3 was adjusted for variables in model 2 plus salt intake, LDL-C, HDL-C, hypertension, diabetes

Model 4 was adjusted for variables in model 3 plus age at menopause

### Sensitivity analyses

After adjustment for variables in the model 2, compared with participants in the Q1 group, the adjusted HR (95% CI) for MI in the highest quartile of baseline TyG index was 2.98 (1.34–6.64). However, there were no significant associations between baseline TyG index and MI, after adjusting for variables in the model 3 or 4. After adjusting for variables in the model 2, 3 or 4, there were a significant association between the highest quartile of TyG index and risk of stroke (Additional file 2: Table S1).

The associations between the highest quartile of baseline TyG index and risk of CVD were similar after excluding participants who received anti-diabetic drugs, lipid-lowering drugs, or antihypertensive drugs (Additional file 2: Table S2).

## Discussion

In the Kailuan study, we observed that higher TyG index at baseline, was associated with higher risk of future CVD and stroke among postmenopausal women regardless age at menopause and obesity status. However, the association between the TyG index and MI was not significant.

Previous several studies revealed that TyG index was associated with an increased CVD risk [20]. And one study indicated stronger association between TyG index and MI risk in females than in males [21]. A prospective cohort study demonstrated higher levels of fasting serum insulin and HOMA-IR were associated with hypertension in women whereas hypertension in men [22]. A protection cardiovascular effect by estrogen decreases and IR increases in postmenopausal women [23]. As a result, monitoring the association between TyG index and risk of CVD in postmenopausal women has implications for the prevention of CVD. Our study found that higher TyG index has significant association with increased risk of future CVD, and this association was attenuated but remained significant after adjusting for HDL-C. The result which derived from a retrospective study including 869 postmenopausal women is similar with our result, and demonstrated that the TyG index may be a predictor of coronary artery disease risk [24]. We found a study including 15,288 postmenopausal women who had a median age of 64 years showed HOMA-IR remained associated with high risk of CVD after adjusting for most CVD risk factors, whereas this association was not significant after adjusting for HDL-C (HR, 1.06; 95% CI 0.98–1.15) [7]. The possible cause that our study differs from our results was racial and residence difference. Thus, our findings might be more applicable to population in northern China.

In model 2 of our study, the highest quartile of baseline TyG index was associated with higher risk of developing MI. However, the association between baseline TyG index and risk of MI was not statistically significant in the full model. But the study result indicated the higher the quartile of Tyg index, the higher the HR. A study including 6093 participants from the third National Health and Nutrition Examination Survey demonstrated the TyG index > 9 was associated with increased risk of subclinical myocardial damage (odd ratio [OR] = 1.21, 95% CI 1.03–1.43) [25]. Several other studies have shown that an elevated TyG index at baseline is associated with risk of MI [26, 27]. Onset age of coronary heart disease was later in women than in men [28]. This lack of statistical significance in the full model of study may be the low numbers of MI events. Incidence rate has shown an increasing trend, and continuous follow-up is necessary. Hypertension is a major risk factor for CVD and contributes a larger proportion at baseline 3270 (42.24%), which might be another cause for the lack of statistical significance [29, 30].

A study including 1250 participants without cardiovascular risk factors showed that the TyG index was an independent marker to predict subclinical coronary disease [31]. We found similar results in the participants without hypertension or diabetes. Compared with the lowest quartile of TyG index, participants in the highest quartile of TyG index were at a 102% (95% CI 1.18–3.46), 61% (95% CI 1.12–2.32) higher risk of CVD, respectively. This suggests that even in healthy population, high TyG index might have a significant impact on identifying population at high risk of CVD. And this has important significance for primary prevention of CVD.

The significance of this study is that our study found the TyG index might be recognized as a risk factor for CVD incident. The TyG index as a surrogate marker of IR was closely linked with the onset and progression of risk factors for cardiovascular such as diabetic, dyslipidemia, high baPWV and so on [13, 32]. These findings demonstrated the TyG index can identify individuals who are at high risk of developing CVD and have CV risk factors. Thus, early intervention including lifestyles intervention in high-risk individuals might prevent the onset of future CVD.

The main strengths of this study include large sample community population-based, prospective design, and IR reflected by TyG index. Nevertheless, this study involves some limitations. First, as an observational study, we cannot confirm the causal-relationship between TyG index and risk of CVD. In addition, our study population were postmenopausal northern Chinese women, further studies are needed to confirm our findings in other racial/ethnic or regions populations. Third, we cannot assess IR (HOMA) due to the lack of fasting insulin level, we could not compare the roles of TyG index with HOMA for the development of CVD.

## Conclusion

In conclusion, our study implies that participants with high TyG index are at a higher risk of developing CVD among postmenopausal women. Our findings indicate the TyG index may serve as a simple and easy marker for early identification of high-risk individuals in the postmenopausal women. Lifestyle changes including physical exercise may be useful for prevention CVD incidence and development in high-risk individuals, which may reduce the burden on societies and families.

## Supplementary Information

**Additional file 1: Figure S1** Flowchart of the study.

**Additional file 2: Table S1.** Association between TyG index with MI and stroke among postmenopausal women. **Table S2.** Association between TyG index with cardiovascular disease among postmenopausal women in sensitivity analysis.

## Data Availability

The datasets used and/or analyzed during the current study are available from the corresponding author on reasonable request.

## Abbreviations

BMI: Body mass index
CVD: Cardiovascular disease
CI: Confidence interval
DBP: Diastolic blood pressure
FBG: Fasting blood glucose
Hs-CRP: High sensitive C-reactive protein
HR: Hazard ratio
HOMA-IR: Homeostasis model assessment of IR
HDL-C: High density lipoprotein cholesterol
IR: Insulin resistance
LDL-C: Low density lipoprotein cholesterol
MI: Myocardial infarction
OR: Odd ratio
SD: Standard deviation
SBP: Systolic blood pressure
TC: Total cholesterol
TG: Triglyceride
TyG: Triglyceride-glucose
