# Supplementary figures and images for: Association between triglyceride-glucose index and risk of cardiovascular disease among postmenopausal women
Source: Cardiovasc Diabetol. 2023 Jan 30;22:21. doi: 10.1186/s12933-023-01753-3 (PMC9887910; doi:10.1186/s12933-023-01753-3)

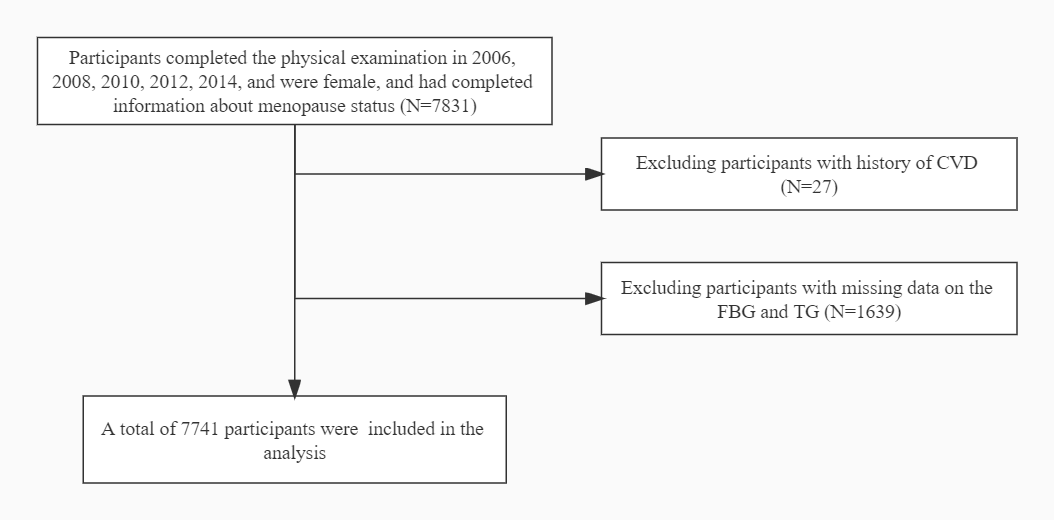

Supplement: Supplementary file 1 — Additional file 1: Figure S1 Flowchart of the study. [file 12933_2023_1753_MOESM1_ESM.jpg]
