# Supplementary material for: Association between triglyceride-glucose index and risk of cardiovascular disease among postmenopausal women
Source: Cardiovasc Diabetol. 2023 Jan 30;22:21. doi: 10.1186/s12933-023-01753-3 (PMC9887910; doi:10.1186/s12933-023-01753-3)
Supplement: Supplementary file 2 — Additional file 2: Table S1. Association between TyG index with MI and stroke among postmenopausal women. Table S2. Association between TyG index with cardiovascular disease among postmenopausal women in sensitivity analysis. [file 12933_2023_1753_MOESM2_ESM.docx]

**Table S1** Association between TyG index with MI and stroke among postmenopausal women

| Outcomes | Quartiles of TyG index | | | | P for trend | Per 1 unit increase |
| --- | --- | --- | --- | --- | --- | --- |
|  | Q1 | Q2 | Q3 | Q4 |  |  |
| MI | | | | | | |
| Case, n(%) | 8(0.40) | 11(0.57) | 16(0.84) | 30(1.56) |  |  |
| Incidence, per 1000 person-y | 0.36 | 0.51 | 0.73 | 1.36 |  |  |
| Model 1 | Reference | 1.34(0.54-3.34) | 1.82(0.78-4.26) | 3.32(1.52-7.25) | <0.01 | 2.00(1.45-2.75) |
| Model 2 | Reference | 1.26(0.50-3.14) | 1.66(0.70-3.93) | 2.98(1.34-6.64) | <0.01 | 1.93(1.39-2.69) |
| Model 3 | Reference | 1.14(0.45-2.85) | 1.31(0.55-3.15) | 1.76(0.74-4.16) | 0.15 | 1.41(0.95-2.09) |
| Model 4 | Reference | 1.13(0.45-2.84) | 1.31(0.55-3.14) | 1.76(0.75-4.17) | 0.15 | 1.42(0.96-2.10) |
| Stroke | | | | | | |
| Case, n(%) | 47(2.36) | 67(3.50) | 78(4.08) | 131(6.82) |  |  |
| Incidence, per 1000 person-y | 2.12 | 3.16 | 3.62 | 6.08 |  |  |
| Model 1 | Reference | 1.44(0.99-2.09) | 1.58(1.10-2.27) | 2.61(1.87-3.65) | <0.01 | 1.67(1.44-1.95) |
| Model 2 | Reference | 1.33(0.92-1.94) | 1.42(0.99-2.06) | 2.27(1.61-3.20) | <0.01 | 1.57(1.34-1.84) |
| Model 3 | Reference | 1.30(0.89-1.90) | 1.29(0.89-1.88) | 1.81(1.26-2.61) | <0.01 | 1.34(1.12-1.60) |
| Model 4 | Reference | 1.30(0.89-1.90) | 1.29(0.89-1.88) | 1.81(1.26-2.61) | <0.01 | 1.34(1.12-1.60) |

Model 1 was adjusted for age at baseline.

Model 2 was adjusted for variables in model 1 plus BMI, education status, current drinker, current smoker, physical activity at baseline.

Model 3 was adjusted for variables in model 2 plus salt intake, LDL-C, HDL-C, hypertension, diabetes.

Model 4 was adjusted for variables in model 3 plus age at menopause.

**Table S2** Association between TyG index with cardiovascular disease among postmenopausal women in sensitivity analysis

| Group | Model 2 |  | Model 3 |  | Model 4 |
| --- | --- | --- | --- | --- | --- |
|  | HR(95%CI) |  | HR(95%CI) |  | HR(95%CI) |
| Excluding participants with anti-diabetic drugs(n=7446). | | | | | |
| Q1 | Reference |  | Reference |  | Reference |
| Q2 | 1.34(0.94-1.90) |  | 1.27(0.89-1.82) |  | 1.27(0.89-1.82) |
| Q3 | 1.49(1.05-2.10) |  | 1.29(0.91-1.83) |  | 1.29(0.91-1.83) |
| Q4 | 2.30(1.66-3.19) |  | 1.66(1.17-2.34) |  | 1.66(1.17-2.35) |
| Excluding participants with anti-diabetic drugs, lipid-lowering drugs(n=7340). | | | | | |
| Q1 | Reference |  | Reference |  | Reference |
| Q2 | 1.36(0.95-1.94) |  | 1.30(0.91-1.86) |  | 1.30(0.91-1.86) |
| Q3 | 1.52(1.07-2.15) |  | 1.32(0.93-1.89) |  | 1.32(0.93-1.89) |
| Q4 | 2.35(1.69-3.27) |  | 1.70(1.20-2.42) |  | 1.70(1.20-2.42) |
| Excluding participants with anti-diabetic drugs, lipid-lowering drugs, or antihypertensive drugs(n=6393). | | | | | |
| Q1 | Reference |  | Reference |  | Reference |
| Q2 | 1.38(0.92-2.06) |  | 1.37(0.91-2.06) |  | 1.37(0.91-2.06) |
| Q3 | 1.59(1.07-2.37) |  | 1.45(0.97-2.18) |  | 1.45(0.97-2.18) |
| Q4 | 2.43(1.67-3.54) |  | 1.72(1.14-2.58) |  | 1.72(1.14-2.58) |
| Excluding participants with diabetes or dyslipidemia (n=6113). | | | | | |
| Q1 | Reference |  | Reference |  | Reference |
| Q2 | 1.33(0.92-1.91) |  | 1.29(0.89-1.86) |  | 1.29(0.89-1.86) |
| Q3 | 1.50(1.04-2.15) |  | 1.41(0.98-2.03) |  | 1.41(0.98-2.03) |
| Q4 | 1.81(1.25-2.62) |  | 1.76(1.21-2.55) |  | 1.76(1.22-2.56) |

Model 2 was adjusted for age, BMI, education status, current drinker, current smoker, physical activity at baseline.

Model 3 was adjusted for variables in model 2 plus salt intake, LDL-C, HDL-C, hypertension, diabetes.

Model 4 was adjusted for variables in model 3 plus age at menopause.
